# Fine-mapping and association analysis of candidate genes for papilla number in sea cucumber, *Apostichopus japonicus*

**DOI:** 10.1007/s42995-022-00139-w

**Published:** 2022-08-23

**Authors:** Xinghai Zhu, Ping Ni, Marc Sturrock, Yangfan Wang, Jun Ding, Yaqing Chang, Jingjie Hu, Zhenmin Bao

**Affiliations:** 1 Ministry of Education Key Laboratory of Marine Genetics and Breeding, College of Marine Life Sciences, Ocean University of China, Qingdao, 266003 China; 2 Department of Physiology and Medical Physics, Royal College of Surgeons in Ireland, Dublin, D02 YN77 Ireland; 3 College of Fisheries and Life Science, Dalian Ocean University, Dalian, 116023 China; 4 Laboratory of Tropical Marine Germplasm Resources and Breeding Engineering, Sanya Oceanographic Institution, Ocean University of China, Sanya, 572000 China

**Keywords:** Genome-wide association study, *Apostichopus japonicus*, Molecular breeding, Candidate genes, Papilla number

## Abstract

The papilla number is one of the most economically important traits of sea cucumber in the China marketing trade. However, the genetic basis for papilla number diversity in holothurians is still scarce. In the present study, we conducted genome-wide association studies (GWAS) for the trait papilla number of sea cucumbers utilizing a set of 400,186 high-quality SNPs derived from 200 sea cucumbers. Two significant trait-associated SNPs that passed Bonferroni correction (*P* < 1.25E−7) were located in the intergenic region near *PATS1* and the genic region of *EIF4G*, which were reported to play a pivotal role in cell growth and proliferation. The fine-mapping regions around the top two lead SNPs provided precise causative loci/genes related to papilla formation and cellular activity, including *PPP2R3C*, *GBP1*, and *BCAS3*. Potential SNPs with *P* < 1E−4 were acquired for the following GO and KEGG enrichment analysis. Moreover, the two lead SNPs were verified in another population of sea cucumber, and the expressive detection of three potential candidate genes *PATS1*, *PPP2R3C*, and *EIF4G* that near or cover the two lead SNPs was conducted in papilla tissue of TG (Top papilla number group) and BG (Bottom papilla number group) by qRT-PCR. We found the significantly higher expression profile of *PATS1* (3.34-fold), *PPP2R3C* (4.90-fold), and *EIF4G* (4.23-fold) in TG, implying their potential function in papilla polymorphism. The present results provide valuable information to decipher the phenotype differences of the papilla trait and will provide a scientific basis for selective breeding in sea cucumbers.

**Supplementary Information:**

The online version contains supplementary material available at 10.1007/s42995-022-00139-w.

## Introduction

The economical mariculture species sea cucumber *Apostichopus japonicus* is subordinated to the phylum Echinodermata, primarily cultured in the coastal regions of Russia, Japan, and northern China due to its rich nutrient and considerable medicinal value (Han et al. 2016; Lv et al. 2022; Toralgranda et al. 2008). The papilla of sea cucumber is not only a multifunctional organ that performs respiration and contact with the external environment, but also a commercially important trait in marketing, i.e., the length and number (Ru et al. 2019). The sea cucumber individuals, which grew more papilla, were deemed as first quality and more valuable in China. To accord with the demands of the market, two new strains of sea cucumber “Shuiyuan NO.1” and “Anyuan NO.1” with increased papilla numbers, i.e., 40.0% and 12.6%, were generated by morphology selection (Rural Economic Committee of Jinzhou 2014; Song and Wang 2019). However, the molecular mechanism behind this trait is still unclear. Genetic-based molecular breeding aiming at papilla number is of great significance to sea cucumber aquaculture.

To date, no comprehensive investigation of the candidate genes of papilla has been made, although previous research primarily focused on morphology, immunology, and transcriptomics in *A. japonicus* (Chen et al. 2020a, b; Deng et al. 2008; Purcell et al. 2012). Studies have shown that the Russian population has the largest total number of papilla than the other eight different *A. japonicus* populations (Chang et al. 2011). On the aspect of the immune response, the *AjAIF1* (*allograft inflammatory factor 1*) was significantly up-regulated after the papilla injury experiment, and it may facilitate wound healing (Ji et al. 2014). Using RNA-seq technology, comparative transcriptome analysis between two important organs, i.e., skin and papilla, unveiled 288 papilla-specific genes that mainly participate in collagen synthesis, ribosome pathway, and tight junction, in which the pinpointed PP2A (Serine/threonine protein phosphatase) could contribute to morphological differentiation (Zhou et al. 2016). Moreover, a similar result has been reported in transcriptomes and digital gene expression analysis of pentactulas (without papillae) and juveniles (with papillae) of *A. japonicus* (Zhan et al. 2019). However, because of lacking systematic screening throughout the genome, the pivotal loci/genes affecting the papilla number of *A. japonicus* remain obscure. Genome-wide association study (GWAS), which is deemed as a universal tool to determine genetic variants analyzing complex traits (Korte and Ashley 2013), has been extensively implemented for growth-related traits or tolerance-related traits in economically important marine animals, such as fish (Gutierrez et al. 2015), shrimp (Khor et al. 2018), crab (Hui et al. 2017), abalone (Yu et al. 2021) and scallop (Zhu et al. 2021; Zeng et al. 2022), using high-density of markers. In *A. japonicus*, GWAS merely conducted in identifying SNPs associated with various body colors (Ge et al. 2020); however, none have been reported to date for papilla number polymorphism.

In the present study, we focused on identifying SNP markers significantly associated with the papilla trait of sea cucumber by performing GWAS combined with the fine-mapping method (Fang and Georges 2016). Further verification of the lead SNPs was carried out in another population, followed by applying the Gene Ontology (GO) and Kyoto Encyclopedia of Genes and Genomes (KEGG) as complementary to the mini-effect genes that interacted with the papilla number trait. Moreover, the expressions of most-potential candidate genes were detected to investigate their critical roles involved in target traits. The SNP-based heritability of the papilla was estimated as one of the genetic parameters for economically important traits in selective breeding programs. The results of this study will provide candidate loci/genes for promoting the genetic and molecular breeding development of *A. japonicus*.

## Results and discussion

### Phenotypes and genotypes

**Fig. 1:**
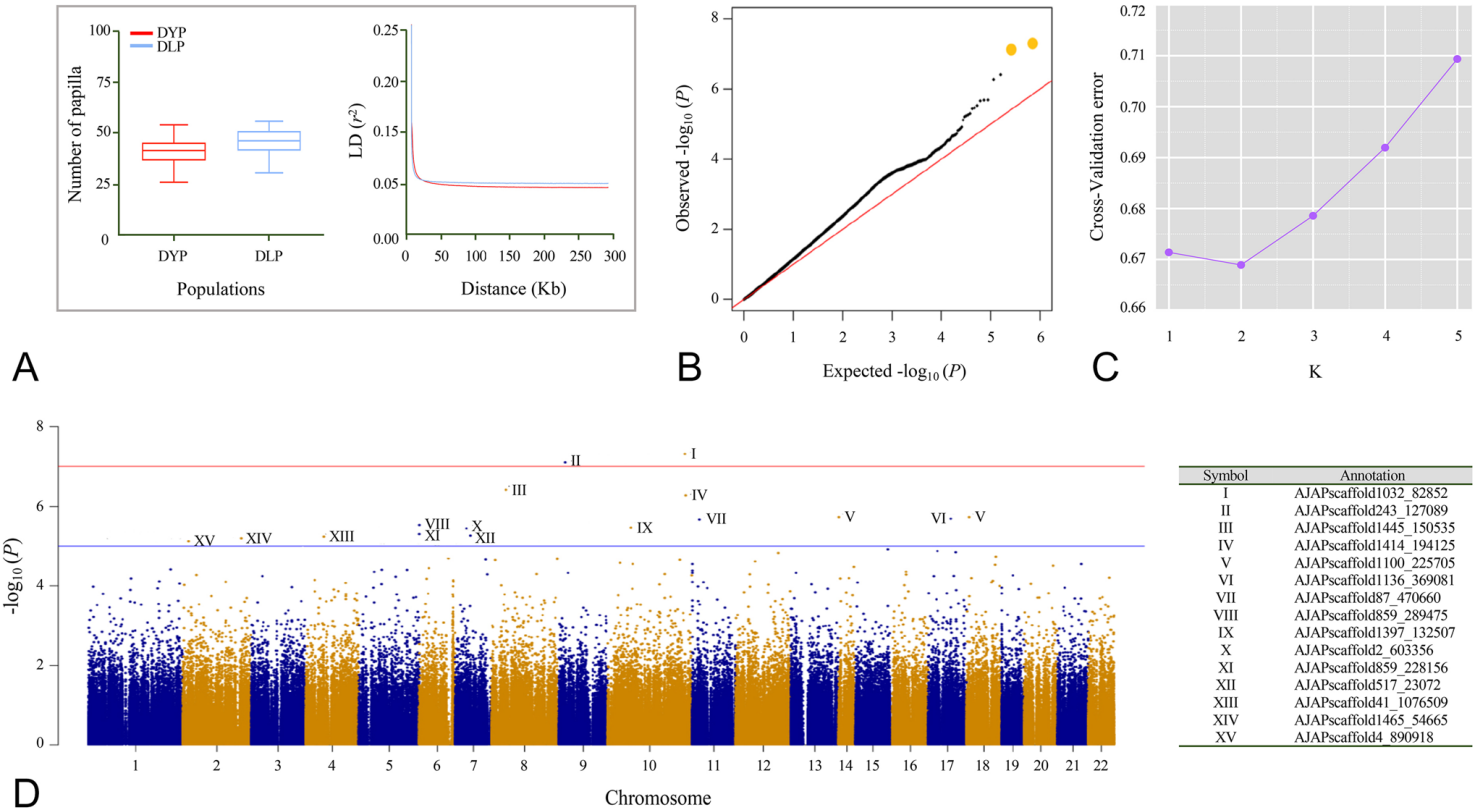
The phenotypic and genetic analysis of papilla number trait. **A** Papilla number (left) and linkage disequilibrium (LD) decay analysis (right). **B** Quantile–quantile (QQ) plots. **C** Genetic clustering analysis. **D** The Manhattan plots for papilla trait in the sea cucumber, where the red line indicates the Bonferroni cut-off (*P* < 1.25E−7) and the blue line indicates the conventional threshold (*P* < 1E−5)

Benefiting from its rich nutritional value, the echinoderm of sea cucumber (*A*. *japonicus*) had a significant share of aquaculture in northern China (Tian et al. 2017). Papilla number is a commercial target trait that varied from geographic sites and breeds (Song and Wang 2019). This study aims to perform GWAS analysis to unveil papilla number-related SNPs and to dredge up candidate genes. The phenotypic trait is the first analysis. As previous research documented in Asian populations, the largest total number of sea cucumber papilla was measured from the Russian population (66.25 ± 8.11), followed by the Japanese population (53.64 ± 7.88) and two Chinese hybrid populations (Chinese–Russian: 54.65 ± 4.74 and Chinese–Korean: 46.29 ± 6.91), whereas the lowest number was 29.57 ± 3.31 of Chinese wild population (Chang et al. 2011). In this study, the 200 sea cumbers animals consisted of two populations DYP (30) and DLP (170), and records showed the papilla number of DYP (36.85 ± 5.74) is significantly less (*P* < 0.01) than DLP (40.70 ± 6.36) (Fig. 1A) and satisfy the normality, which is regarded as pre-condition of GWAS (Supplementary Fig. S1). Unlike SNP-chip unguaranteed quality (Talouarn et al. 2020) or restriction-site-associated DNA sequencing (RAD-seq) limited coverage (Xu et al. 2019), we adopted the whole-genome sequencing method to obtain most SNPs to enhance the accuracy and power of GWAS (Wu et al. 2019a, b). In a summary of sequenced data, each genomic library generated 37 million 350-bp paired-end reads (~ 10.42 X coverage) that were aligned to the *A. japonicus* reference genome (Zhang et al. 2017). To assemble chromosome-scale scaffolds, we used the published linkage maps (Tian et al. 2015) inferred from 2b-RAD markers (Wang et al. 2012) and the *A. japonicus* reference genome (Zhang et al. 2017) to linearly arrange contigs. Approximately 87% of the total reads were aligned to the reference genome and used for SNP calling. After rigid control (call rate > 90%, MAF < 0.05), a set of 400,186 high-quality SNPs were captured for downstream analysis.

### Genetic structure and parameter analysis

The crucial factor in selective breeding is to determine the proportion of phenotypic variance explained by genetic variance (Falconer and Mackay 1996). First, we applied the GCTA method to determine the potential correlation between genetic and phenotypic similarities, partitioning the total phenotypic variance into genetic and environmental factors (Yang et al. 2011). There are three algorithms of REML in GCTA: average information (AI) (Johnson and Thompson 1995), fisher scoring (FS) and expectation maximization (EM) (Searle et al. 1992). The estimates of SNP-based heritability (${h}_{\mathrm{GCTA}}^{2}$) conducted using all SNPs (400,186 SNPs with MAF > 5%) were 0.66 (S.E. 0.07) by REML AI, 0.65 (S.E. 0.08) by REML FS, and 0.65 (S.E. 0.09) by REML EM, respectively. This result is consistent with the previous findings that SNP-based heritability for growth traits was at higher levels than adaptive traits (tolerance and resistance traits) in aquatic animals, such as shell width traits (0.54) in Zhikong scallop (Guo et al. 2018a), soft tissue weight (0.72) in Pacific abalone (Peng et al. 2021), ammonia tolerance (0.36) in orange-spotted grouper (Shan et al. 2021) and *Vibrio harveyi* resistance (0.38) in yellow drum (Luo et al. 2021). Moreover, on account of Breeder’s equation, given as *R* = *h*^2^ *S* (*R*: response to selection, *S*: selection differential) (Roff 1997), higher heritability may result in a stronger response and more effective selection. Thus, identifying the trait-related markers is of great importance to accelerate the genetic improvement of this commercial trait and promote the development of new breeds. However, the major challenge for association analysis within 200 individuals is the population structure and familial relatedness. It is widely acknowledged that the ignorance of population stratification and kinship may produce mass false positives, and therefore result in unreliable outcomes (Marigorta et al. 2018). Regardless of plants (Müller et al. 2019; Zhong et al. 2021) and animals (Guo et al. 2018b; Higgins et al. 2018), to identify the phenotype-linked causal variants, geographic populations with complex population structures and kinships (also known as Q + K) were collected to fit a mixed liner model. In our results, the lowest cross-validation (CV) error value was *K* = 2 in the admixture analysis (Fig. 1C), which was subsequently exemplified by the PCA analysis (Supplementary Fig. S2), inferring two groups stratified among samples. Most of the kinship coefficients between pairs of individuals were between 0 and 0.2 (Supplementary Fig. S3), suggesting the weak relative kinship within two populations, which was optimized to improve the GWAS power. In addition, for linkage disequilibrium analysis (Fig. 1A), both populations exhibited rapid LD decay, which decreased to 0.2 beyond 62 bp (DLP) and 54 bp (DYP), respectively. The result showed a close genetic relatedness of these samples, indicating downstream association analysis needed a sufficient number of SNPs.

### Genomic regions associated with papilla number

GWAS is widely acknowledged as a powerful approach to reveal the genetic variation linked to complex phenotypes and to identify the candidate hub genes of economic traits, hence providing valuable markers for a breeding program (Luo et al. 2012). Our GWAS results for the papilla number trait in sea cucumber are summarized in Table 1 and Fig. 1D. And the QQ plot (Fig. 1B) indicated a good correction of population stratification, for major SNPs following null hypothesis and only a few deviated significant SNPs. In total, the two strongest signals (AJAPscaffold1032_82852 with *P* < 4.82E−8 and AJAPscaffold243_127089 with *P* < 7.81E−8) that passed the Bonferroni correction (0.05/N_SNP_) were detected on chromosome 10 (~ 43.3 Mbp) and chromosome 9 (~ 4.2 Mbp); the MAF of the lead SNPs were 0.16 and 0.27, each explaining 1.48% and 1.41% for phenotypic variance, respectively. The outcomes testified to a common assumption that quantitative traits are jointly affected by a large number of genes with minor impacts and a small quantity of genes with major impacts insofar as only two associations were obtained from the experiment (Lu et al. 2013). Also, similar GWAS results were reported in quantitative traits analysis of marine mollusk species, namely two growth-related SNPs in scallops (Ning et al. 2019), 12 and 13 Zn/Cu-accumulation-related SNPs in oysters (Meng et al. 2020) and 263 SNPs associated with 10 growth-related traits in abalone (Peng et al. 2021). However, Bonferroni correction was employed to reduce multiple hypothesis error rates, which has, in turn, increased the probability of false negatives (Armstrong 2014). At a conventional and less stringent threshold (*P* < 1E−5) (Coltell et al. 2020), there were 13 more SNPs in association with the target trait, which scattered into Chr. 2, 4, 5, 7, 8, 11, 17, 18. Among these potential causal loci, we focused on the two GWAS regions that were identified as 1–2 Mb regions around the lead SNPs. More precisely, near to the position of AJAPscaffold1032_82852 located at 43,374,494 bp of chromosome 10, marker AJAPscaffold1397_132507 at 12,916,805 bp and AJAPscaffold1414_194125 at 43,815,925 bp were strongly associated for the trait with *P* < 3.42E−6 and *P* < 5.27E−7, respectively. On chromosome 5, two strongly linked markers (AJAPscaffold859_228156 with *P* = 4.98E−6 and AJAPscaffold859_289475 with *P* = 2.95E−6) were on the same scaffold (scaffold859). Moreover, the trait had another three strong signals at 8,076,008 bp of chromosome 8 (AJAPscaffold1445_150535 with *P* = 3.87E−7), 898,147 bp of chomesome18 (AJAPscaffold1100_225705 with *P* = 2.03E−6), 12,740,233 bp of chomesome17 (AJAPscaffold1136_369081 with *P* = 2.06E−6). On two regions at ~ 3.3 Mb and ~ 32.8 Mb of chromosome 2 and two regions at ~ 6.3 Mb and ~ 8.5 Mb of chromosome 7, the highly significant associations of two SNPs (AJAPscaffold4_890918 with *P* = 7.31E−6 and AJAPscaffold1465_54665 with *P* = 6.15E−6) on chromosome 2 and two SNPs (AJAPscaffold2_603356 with *P* = 3.59E−6 and AJAPscaffold517_23072 with *P* = 5.44E−6 on chromosome 7 were detected for the trait, respectively. On chromosomes 4 and 11, two SNPs were significant with the trait. Overall, 15 SNPs were identified to be (*P* = 1E−5) associated significantly with the papilla number trait, which could explain a total of 14.78% of phenotypic variation. Unlike papilla number trait-associated SNPs dispersed in 10 various linkage groups, some SNPs that significantly affected quantitative traits were found in a certain genomic region (Yoshida and Yáñez 2021). For example, the SNPs attributed to the carotenoid concentration trait of “Haida golden scallop” were uniquely identified in linkage group 7 (Wang et al. 2022). Through GWAS analysis of body weight in giant grouper, one causal SNP and with two suggestively associated SNPs were all located in chromosome 18 (Wu et al. 2019a, b). The present result was not consistent with previous studies obtained in previous GWASs of some quantitative traits, indicating that the polymorphism of papilla number may well be determined by a more complex mechanism.

**Table 1:** List of all significant SNPs identified in the GWAS analyses sorted by Pval (Pc1df)

*N*	SNP	CHR	Position	Pc1df	effB	VSNP	VP	VG	VP_SNP_ (%)	VG_SNP_ (%)	MAF
1	AJAPscaffold1032_82852	10	43374494	4.82E−08	− 1.88	0.95	90.51	64.16	1.04	1.48	0.16
2	AJAPscaffold243_127089	9	4265016	7.81E−08	1.52	0.49	90.51	64.16	0.91	1.41	0.27
3	AJAPscaffold1445_150535	8	8076008	3.87E−07	− 1.38	0.65	90.51	64.16	0.72	1.01	0.22
4	AJAPscaffold1414_194125	10	43815925	5.27E−07	− 1.81	0.83	90.51	64.16	0.92	1.30	0.15
5	AJAPscaffold1100_225705	18	898147	2.03E−06	1.67	0.78	90.51	64.16	0.86	1.22	0.17
6	AJAPscaffold1136_369081	17	12740233	2.06E−06	2.06	0.89	90.51	64.16	0.99	1.39	0.12
7	AJAPscaffold87_470660	11	4247077	2.16E−06	0.909	0.35	90.51	64.16	0.39	0.55	0.31
8	AJAPscaffold859_289475	5	34009584	2.95E−06	− 1.14	0.49	90.51	64.16	0.54	0.76	0.25
9	AJAPscaffold1397_132507	10	12916805	3.42E−06	− 1.56	0.68	90.51	64.16	0.75	1.07	0.17
10	AJAPscaffold2_603356	7	6304931	3.59E−06	0.75	0.26	90.51	64.16	0.29	0.41	0.39
11	AJAPscaffold859_228156	5	33948256	4.98E−06	0.67	0.21	90.51	64.16	0.23	0.33	0.41
12	AJAPscaffold517_23072	7	8596937	5.44E−06	− 1.01	0.41	90.51	64.16	0.45	0.64	0.28
13	AJAPscaffold41_1076509	4	10420026	5.77E−06	− 1.52	0.65	90.51	64.16	0.72	1.01	0.17
14	AJAPscaffold1465_54665	2	32856199	6.15E−06	1.98	0.76	90.51	64.16	0.84	1.19	0.11
15	AJAPscaffold4_890918	2	3387562	7.31E−06	1.45	0.64	90.51	64.16	0.71	1.01	0.19

*SNP* the name of the single-nucleotide polymorphism, *CHR* chromosome, *Position* position of the SNP on the chromosome in base pairs on the sea cucumber genome, *Pc1df* *P* values adjusted for genomic control, *effB* effect of the minor allele (B allele), *VSNP* variance explained by the SNP (calculated as 2pqa2, where p is the frequency of one allele, *q* = 1 − *p* is the frequency of the second allele and a is the additive genetic effect); *VP* phenotypic variance, *VG* additive genetic variance, *VP*_*SNP*_*(%)*percentage of phenotypic variance explained by each *SPN*, *VG*_*SNP*_* (%)*percentage of additive genetic variance explained by each *SPN*, *MAF* minor allele frequency

### Fine-mapping and candidate genes

For further refinement of the regions containing causative genes and variants, we achieved fine-mapping of the GWAS region 2–3 Mb around the lead SNPs. The advantages of the fine-mapping are that (1) it narrows down potential causative variants by indicating causal variants in the SNP set; and (2) it efficiently identifies more than one variant if multiple variants control the investigated trait. Thus, the fine-mapping could increase the reliability for the GWAS to find the candidate genes (Schaid et al. 2018). To increase the accuracy of fine-mapping, we used as many SNPs as possible by utilizing re-sequenced SNPs with an average coverage of ∼ 10.42×. Then, we narrowed down the clusters containing causative variants using BayesFM-MCMC software.

The software first clustered the SNPs within a GWAS region using a hierarchical clustering method based on the *r*^2^ among SNPs; then it searched multiple causal variants by conducting a Bayesian model selection across the cluster and generated the posterior probability for each SNP within the cluster, from which a credible set of SNPs with > 95% posterior probability was constructed.

**Fig. 2:**
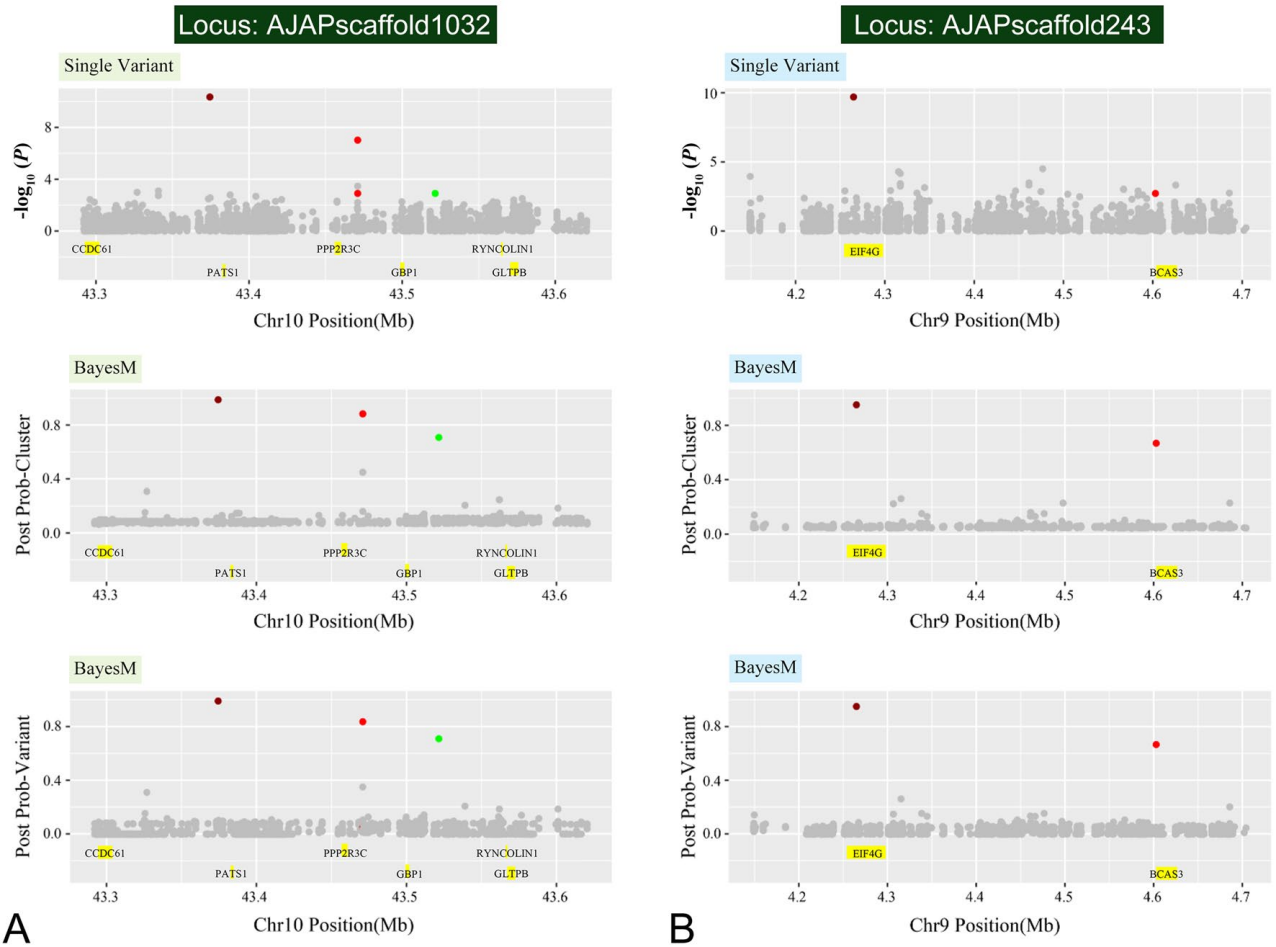
Fine-mapping result of the top two causative SNPs. **A** Fine-mapping the chromosome 10: region. **B** Fine-mapping in the chromosome 9: region

To begin, a single variant association was conducted for the GWAS chromosome region, the scaffold AJAPscaffold1032, which included the significantly associated SNP AJAPscaffold1032_82852. BayesFM-MCMC was used to further refine the region, and three cluster signals were identified with posterior probabilities bigger than 0.8. Most SNPs had minuscule posterior probabilities and three SNPs gave substantial posterior probability (f.i. greater than 0.5) in the identified cluster (Fig. 2A). Fine-mapping of the region on the scaffold AJAPscaffold243, including the significantly associated marker AJAPscaffold243_127089, identified two cluster signals with a posterior probability equal to 1. Most SNPs had minuscule posterior probabilities, and two SNPs gave substantial posterior probability (f.i. greater than 0.5) in the identified cluster (Fig. 2B).

The 3 SNPs of interest on AJAPscaffold1032 are located in the intergenic region, which is approximately 90 kb downstream of *PATS1* (serine threonine protein kinase pats 1), over 20 kb downstream of *PPP2R3C* (serine threonine protein phosphatase 2A regulatory subunit B subunit gamma), and about 30 kb downstream of *GBP1* (guanylate binding protein 1). All three candidate genes have been demonstrated to be involved in the cell process. Specifically, *PATS1* serves as an essential regulator of cytokinesis involved in the binding to actomyosin cytoskeleton. Moreover, disruption of the *PATS1* locus could cause cytokinesis suspension, and therefore was large and multinucleate (Abysalh et al. 2003). *PPP2R3C*, is also known as *G5PR*. One of the members of the PP2A regulatory B subunits has been extensively reported as interacting directly with protein phosphatase PP2A and PP5 (Katayama et al. 2014). The latter are functions in cell cycle progression, cell division, development and cellular signal transductions (Janssens and Goris 2001; Kono et al. 2002; Nolt et al. 2011). As for another candidate gene *GBP1*, substantial evidence exemplifies the association with intestinal and vascular epithelial cell proliferation in humans (Capaldo et al. 2012; Guenzi et al. 2001). We assume that the variants possibly have regulatory effects on the three genes nearby. Therefore, alteration of cellular activities potentially affects the phenotypic trait, which is noticed as papilla number diversity. Furthermore, the two highly linked SNPs on AJAPscaffold243 are located within two genes, *EIF4G* (eukaryotic initiation factor 4G) and *BCAS3* (breast carcinoma amplified sequence 3). *EIF4G* provides a docking site for *Mnk1* (mitogen-activated protein kinases 1) to phosphorylate *EIF4E*, which is an essential modulator of cell growth and proliferation (Pyronnet et al. 1999). Even though without a means to participate cell proliferation process, *BCAS3* is more characterized by promoting directional cell migration (Shetty et al. 2018). Taken together, the functional genes were searched for near the tightly linked regions as candidate genes. Subsequently, function analysis of candidate genes was conducted, revealing most of the genes involved in cellular activities, which may lead to polymorphism of papilla number.

### Gene-set enrichment and pathway-based analysis

**Fig. 3:**
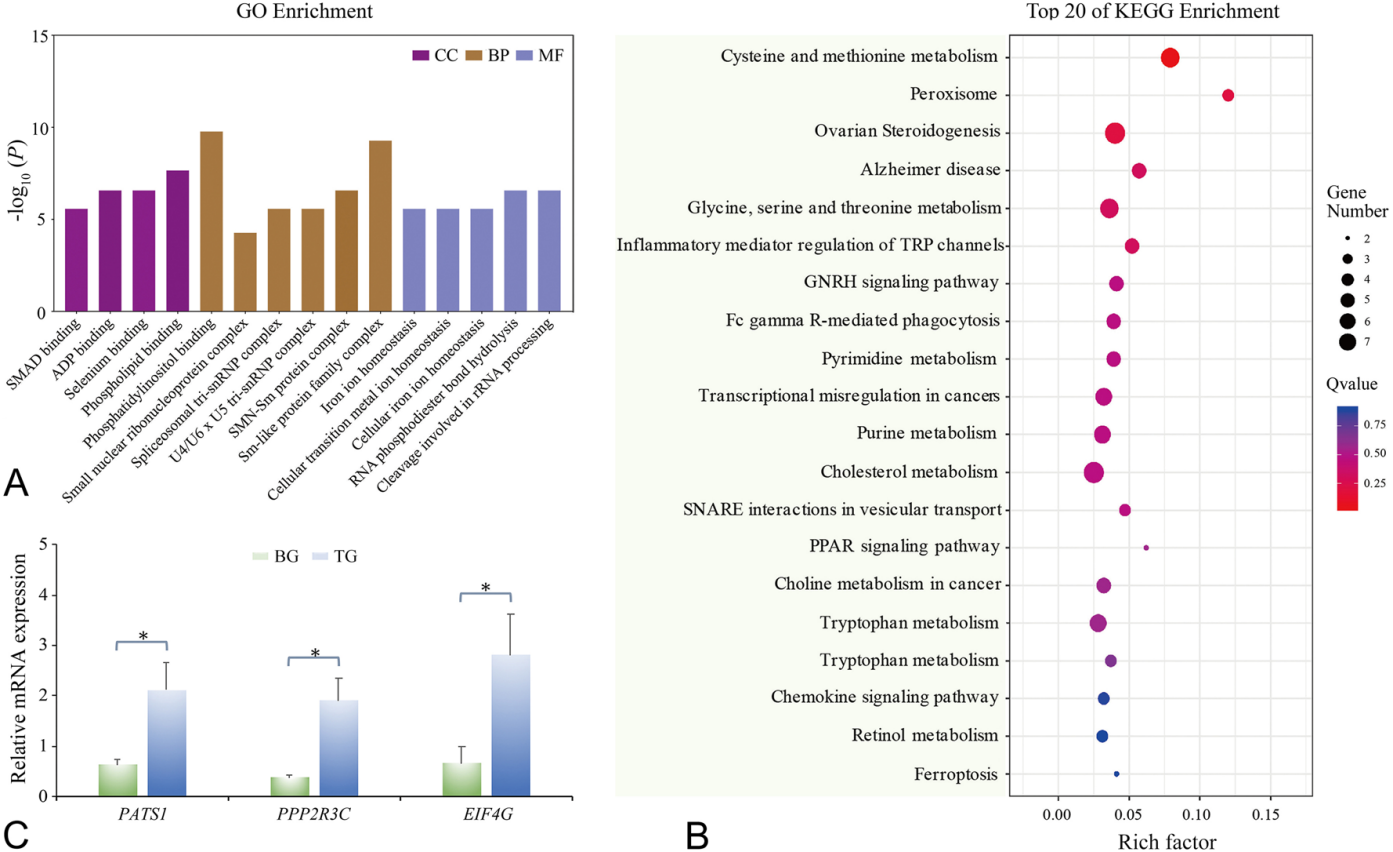
The bioinformatic enrichment and expression analysis of candidate genes. **A** The summary of GO function annotation analysis and each term described the function of gene cluster and the length of colored bars represent the *P* value. **B** The summary of KEGG pathway enrichment analysis and the bubble size and color represented gene numbers and *Q* values in certain pathway. **C** Relative expression levels of *PATS1*, *PPP2R3C* and *EIF4G* in papilla of TG and BG sea cucumbers. Three replicates were performed for each papillae tissue, and three technical replicates were conducted for each PCR. The comparison of the expression levels as performed using an independent sample *t* test. ‘*’ indicates differences that are statistically significant (*P* < 0.05)

At a less stringent significance level (*P* < 1E−4), there were 254 SNPs annotated in 76 genes. After screening the 15 kb window up- or downstream of these significant SNPs, a set of 371 genes in association with papilla number diversity were captured for GO function annotation and KEGG pathway enrichment analyses. This bioinformatic analysis was broadly employed in omics data to speculate potential gene phenotypes (Zhang et al. 2013) and uncover important molecular mechanisms (Feng et al. 2020). In the present study, GO analysis showed 109 genes were successfully mapped to the background (10,328 genes), and the first 5 GO terms with lower *P* value in each module were displayed in Fig. 3A. The most remarkable terms were phosphatidylinositol binding (GO:0035091) in MF, Sm-like protein family complex (GO:0120114) in CC, and cleavage involved in rRNA processing (GO:0000469) in BP, which was similar to previous research on cell growth. For example, phosphatidylinositol 3-(PI3) kinase was proved to interact with transforming growth factor (TGF) β receptors in epithelial cells (Jae et al. 2005). Some Sm and Sm‐like proteins bind to snRNPs (small nuclear ribonucleoprotein) and play a major role in snRNP biogenesis and transport, which mediated cell growth and development with corresponding transcription factors (Séraphin 1995; Xue et al. 2010). Endonuclease cleavage initiated internal transcribed spacer 1 (ITS1) to separate rRNA components of ribosomal subunits, production of which is coupled to the cellular growth rate (Sloan et al. 2013). This result is in line with the formation of papillae, which were composed of inner mesothelium, nerve plexus, the outer epidermis, and connective tissue layer (Vandenspiegel et al. 1995). This suggests that the higher expression of cell growth-related genes could result in greater papilla number of *A*. *japonicus*, especially for the genes functioned in skins cells. Besides, KEGG pathway enrichment analysis (Fig. 3B) was complementary to the process affecting the papilla number trait. Target genes were principally classified into three classes, amino acid, and nucleotide metabolism, endocrine system and human diseases, among which purine (KO00230) and pyrimidine (KO00240) metabolism was involved in fibroblast cell proliferation (Engström and Zetterberg 1984). Therefore, this may well influence the growth of connective tissue during papillae development (Mondain and Ryan 1993). Another two significantly enriched categories, PPAR (peroxisome proliferator-activated receptor) signaling pathway (KO03320) and GnRH (gonadotropin-releasing hormone receptor) signaling pathway (KO04912) were enrolled in the endocrine system. Activation of hepatic stellate cells could up-regulate platelet-derived growth factor-*β* receptor and epidermal growth factor receptor; meanwhile, down-regulating *PPAR-γ* expression, the latter of which was a crucial factor in fat cell differentiation (Zhou et al. 2007), with no exception to papilla components. The GnRH signaling pathway, which was able to activate MAPKs to transfer signals from the cell surface to the nucleus and affected gonadotropin transcription (Kraus et al. 2001), suggested that GnRH may be responsible for the cell proliferation cycle. These results implicate that papilla number diversity in sea cucumber may be due to the activities involved in cell growth. Such biological processes were not only dominated by multiple loci/genes, but also modified by a great number of related minor loci/genes.

**Table 2:** Sequences of primers in this study

SNP/gene	Primers sequence	Note
AJAPscaffold1032_82852	F:ATCCTTGCCAACAAACTTCT	SNP verification
	R:TGGGACGAATGAGGAGAGGT	
AJAPscaffold243_127089	F:CTCCACCCTTTTCTTTCCCTTA	SNP verification
	R:TCAAAATGGGCAAATTGCTATC	
*AjPATS1*	F:GCCGCTCAGGATCAGGATAT	qRT-PCR
	R:ATTCCTCGCTACACCCCTCC	
*AjPPP2R3C*	F:AACAGGTAAAATAAAGATTCAGG	qRT-PCR
	R:ATTTAGATATTGACCATAGACCC	
*AjEIF4G*	F:TGATGCCGCCAGCAATGTAT	qRT-PCR
	R:TTTGTGGTGGCTGCGTTTGC	
*β-actin*	F: AAGGTTATGCTCTTCCTCACGC	Inter control gene
	R: GATGTCACGGACGATTTCACG	

### SNP verification and candidate genes evaluation

The top two SNPs (AJAPscaffold1032_82852 and AJAPscaffold243_127089) that respectively located in intergenic and intron regions, were identified by GWAS, and were successfully verified via Sanger sequencing based on the primers in Table 2 within the group TG (with more papilla number, 41.74 ± 2.57) and the group BG (29.13 ± 2.39, with less papilla number) from another sea cucumber population. The allele and genotype frequency of the two SNPs are presented in Table 3. For SNP, AJAPscaffold1032_82852, genotype TT was only observed in the group TG, with genotypes GT (66.67%) and GG (6.7%), whereas genotypes GG (80%) were mainly called in the group BG and genotypes GT for the remaining 20%. A similar result was found for SNP AJAPscaffold243_127089 (for AA, AG, GG; 0, 46.67, 53.33% in the group TG and 20, 73.33, 6.67% in the group BG, respectively). Comparison between individuals in the group TG and BG showed significant differences (*P* < 0.05) in genotype frequencies of the top two SNP markers. Furthermore, the gene expression patterns of the candidate genes were detected in the papilla tissue of 20 healthy individuals using the qRT-PCR method. As shown in Fig. 3C, *PATS1*, *PPP2R3C* and *EIF4G* of individuals in group TG consistently and significantly were expressed more so than in the group BG with approximately 3.34-, 4.90-, 4.23-fold, respectively. These results support the evidence that the candidate loci/genes may well be involved in the papilla number diverse of the individuals in groups TG and BG.

**Table 3:** Comparison of genotype frequencies of the top two SNPs between group TG (*N* = 15) and group BG (*N* = 15) of sea cucumber

SNP	Location	Locus	Genotype	Number of animals		Fisher’s exact test *P* value
				TG	BG	
AJAPscaffold1032_82852	Intergenic	T < G	TT	4	0	0.0001124
			GT	10	3	
			GG	1	12	
AJAPscaffold243_127089	Intron	A < G	AA	0	3	0.00773
			AG	7	11	
			GG	8	1	

The top 15 and the bottom 15 sea cucumbers were selected based on their papilla number and tagged as TG (Top group) and BG (Bottom group)

Although the top two SNPs did not locate in the coding region, they could affect trait-related functional genes by transcriptional regulation (Sturm et al. 2008; Visser et al. 2014). The significant genotype differences (*P* < 0.01) were found in two comparative groups, TG and BG, based on papilla number. Meanwhile, the expressions of three candidate genes showed significant differences (*P* < 0.05), which validated the reliability of the two SNPs and in accord with the consensus that functional genes existed around the significant SNP (Pharoah et al. 2013). Specifically, our result showed the mRNA expressional level of *PATS1* in TG was over three times that in BG (*P* < 0.05), indicating *PATS1* might function in papilla formation. As reported, disrupted *PATS1* locus resulted in cytokinesis-defective phenotype in *Dictyostelium discoideum* (Abysalh et al. 2003) indicating *PATS1* might influence cytokinesis activity related to papilla formation. Recent research revealed variants in another serine threonine-related protein, PPP2R3C, may well enhance the catalytic activity of PP2A to impair SOX9 (SRY-related HMG-box) signaling and therefore regulate cell development (Sandal et al. 2021). The extremely high expression (4.90-fold) of *PPP2R3C* detected in TG could promote the cell differentiation process during papilla formation to grow more papillae. As for candidate gene *EIF4G*, substantial evidence proved the regulatory function of EIF4G/EIF4E complex in cell proliferation and growth (Moerke et al. 2007). Besides, overexpression of *EIF4G* would induce malignant transformation of normal cells. Thus, we speculated that the higher expression of *EIF4G* (4.32-fold) in TG rather than BG could increase cell proliferation involved in papilla generation. Overall, these combined results may support that the verified two SNPs and their corresponding candidate genes might contribute to the diversity of papilla number of sea cucumber.

## Conclusion

Two domestic populations of *A. japonicus* exhibited diverse papilla numbers with a relative-high SNP heritability (*h*^2^ = 0.65 ± 0.09). Using GWAS analysis, we found two SNPs significantly associated with the papilla number of sea cucumber. Subsequent fine-mapping regions of the two SNPs provided more precise causative loci/genes, *PATS1*, *PPP2R3C* and *EIF4G*, which could affect the papilla number trait. GO function annotation and KEGG pathway enrichment analyses revealed the possible molecular mechanism of the diversified papilla number. The top two SNPs were successfully verified in another population of *A. japonicus*. Meanwhile, the expression levels of the three genes *PATS1*, *PPP2R3C*, and *EIF4G* of animals in the group TG (Top papilla number group) were found to have significantly higher expression profiles with 3.34-fold, 4.90-fold, and 4.23-fold, respectively, compared to its expression levels in the group BG (Bottom papilla number group), suggested their underlying role in growing more papillae. The present results provide valuable information to decipher the phenotype differences of the papilla trait and will provide important reference information and methodology basis for breeding in sea cucumbers.

## Materials and methods

### Animals, phenotypes, and genotypes

The 200 individual animals of sea cucumber used in the present study were comprised of two domestic populations, the DLP (38° 53′ 27″ N, 121° 56′ 35″ E, Dalian, Liaoning Province, China) and DYP (38° 13′ 8″ N, 118° 24′ 36″ E, Dongying, Shandong Province, China), and the number records of papilla number followed Chang’s methods (Chang et al. 2011). Afterward, muscle tissue samples were collected and used for DNA extraction via the CTAB method. Pair-end sequencing libraries with an insert size of 350 bp were constructed for each sample and sequenced on the NovaSeq platform. *FastQC* software (http://www.bioinformatics.babraham.ac.uk/projects/fastqc/) was employed to sequencing reads control. High-quality reads were mapped to the reference genome sequence of *A. japonicus* (Zhang et al. 2017) using *Bowtie2* (version 2.3.4.1) with default parameters (Langmead and Salzberg 2012). Alignment data were processed with *Samtools* (version0.1.19) (Li et al. 2009) and *Picard* (http://broadinstitute.github.io/picard) to mark duplicate reads and estimate the average insert size of the paired-end reads. The Genome Analysis Toolkit (GATK) (McKenna et al. 2010) was used for indel realignment, base-score recalibration, and extraction of reads depth information. The BAM files were imported to *Samtools* to conduct reads sorting and filtering, and SNP calling with high-quality biallelic SNPs. The *PLINK* (version 1.9) software (Chang et al. 2015) was then used for further quality control as follows: (1) individuals with missing rate (-mind) < 0.2; (2) SNPs with missing rate (-geno) < 0.1; (3) minor allele frequency (-maf) > 0.05.

### Population structure and genetic parameters calculation

The population structure of the 200 animal samples was evaluated using *Admixture* software (version 1.3) (Alexander et al. 2009) and the number of subgroup (K) was set from 1 to 5. To visualize familial relatedness more precisely, the result of *Tassel* software (version 5) (Bradbury et al. 2007) was delineated by *TBtools* (version 1.0) (Chen et al. 2020a, b), respectively. Besides, genome-wide Complex Trait Analysis (*GCTA*, version 1.93.2) (Yang et al. 2011) was used to estimate the SNP-based heritability of papilla number trait. First, population structure was considered in heritability estimation, then we estimated univariate heritability of papilla number trait of sea cucumber by the restricted maximum likelihood method. Moreover, linkage disequilibrium (LD) statistics and LD decay analysis of both populations were performed using *PopLDdecay* software (version 3.41) with default parameters (Zhang et al. 2019), and the LD decay plot based on mean *r*^2^ was drawn using a Perl script embedded in the software.

### Genome-wide association study and candidate gene exploring

GenABEL-package is R library for facilitating Genome-Wide Association Study (GWAS) analysis of binary and quantitative traits (Aulchenko et al. 2007). We used the Genome-Wide Association using Mixed Model and Regression-Genomic Control (GRAMMAR-GC) approach, with the default function gamma (Amin et al. 2007; Svishcheva et al. 2012), to fit a single marker regression for GWAS.

1 $${y}={X}{{\beta}}+{Z}{{a}}+{M}{{g}}+\epsilon ,$$

where ${y}$ is a vector containing a quantitative trait measured on individual $i$; ${{\beta}}$ is a vector of the fixed effects; ${X}$ is a design matrix, which relates records to fixed effects ${{\beta}}$; ${{a}}$ is a vector of random additive genetic effects with the multi-normal distribution ${{a}}\sim {{N}}\left(0, {G}{\sigma }_{a}^{2}\right),$ where *G* is the genomic relationship matrix (VanRaden 2008) and ${\sigma }_{a}^{2}$ is the additive genetic variance; *g* is the SNP additive effect; ${Z}$ and *M* are the incidence matrices for ${{a}}$ and *g*, respectively; and ${\epsilon }\sim {{N}}(0, {{I}\upsigma }_{{e}}^{2})$, where ${\sigma }_{e}^{2}$ is the residual variance and *I* is an identity matrix. The GRAMMAR-GC method incorporates the ideas underlying structured association and the genomic kinship matrix. Basically, it allows for structured association, using genomic data to identify strata and more subtle structure. Bonferroni correction with a 0.05 cut-off (*P* < 1.25E−7) was set as a stringent threshold, and the conventional threshold of *P* value (1.00E−5) was also considered to declare significances (Coltell et al. 2020). Both Manhattan plots for GWAS results and QQ plot that expressing the expected and observed *P* values were obtained by the R package ‘QQman’ (Turner 2014).

To identify weaker but related signals that were missed owing to the stringency in *P* value thresholds, we used pathway enrichment analyses according to the GWAS results. In the enrichment analysis, the total SNPs tested in GWAS represented the background SNP, whereas the background genes were the genes associated with those SNPs. The SNPs were assigned to genes if they were located within the gene or in a flanking region of 15 kb up- and downstream of the gene (Pickrell et al. 2010). The GO (Ashburner et al. 2000) databases and the Kyoto Encyclopedia of Genes and Genomes (KEGG; Ogata et al. 1999) were queried to assign the genes to functional categories. The gene set enrichment analysis was carried out using the online software (http://www.omicshare.com/tools) and *Enrichpipeline* based on the hypergeometric distribution (Huang et al. 2009). GO terms with *P* < 0.05 were considered significantly enriched.

### Fine-mapping

Using the BayesFM-MCMC package (Fang and Georges 2016) to finely map causative variants, more than one set of variant clusters could be detected in each region. The threshold for SNP clustering was set as *r*^2^ = 0.5; the Markov chain length was 600,000 with the first 20,000 discarded (burn-in period). The threshold to declare significance was set at *P* = 1.1E−5, determined by dividing 0.05 by the number of SNPs in the GWAS region. The software is applicable for either genotyped variant dataset or for imputed variant dataset and is also suitable for both case–control study and for continuous trait study.

### SNP verification

To validate the association test result, two lead SNPs (*P* < 1E−8) were selected to verify in the 300 samples randomly collected from another sea cucumber population in the sea area of Hekou District, Dongying, Shandong Province in late Dec. 2021. Then, the top 15 and the bottom 15 sea cucumbers were chosen hinged on their papilla number and tagged as TG (Top group) and BG (Bottom group), and preserved papillae tissue for subsequent verification. The top two SNPs were verified following the method delineated by Zhu et al. (2021) with some modifications (the annealing temperature was 58.3 °C and 54.2 °C for SNP AJAPscaffold1032-82852 and SNP AJAPscaffold243-127089, respectively) and then sequenced by Sangon Biotech (Shanghai, China). The genotypes of target loci were aligned by ClustalW2 multiple alignment program (http://www.ebi.ac.uk/Tools/msa/clustalw2/), and differences in alleles frequencies between the TG and BG were conducted using Fisher’s exact test to confirm the variant. *P* values < 0.05 were considered statistically significant.

### Candidate gene evaluation

To examine the expression profile of genes, which are the top two SNPs annotated or nearby influenced between TG and BG, 10 TG and 10 BG samples from another non-breeding sea cucumber population (*N* > 300) were collected randomly, and the tissue of papillae was carefully sampled in the sea area of Hekou District, Dongying, Shandong Province. Total RNA extraction of papillae was carried out following the method described by Hu et al. (2006), and the Moloney murine leukemia virus (MMLV) reverse transcriptase (Thermo, Wilmington, USA) was used to acquire cDNA. β-actin (ACTB) was selected as a reference for tissue samples (Wang et al. 2017). Once complete real-time quantitative reverse transcription PCR (qRT-PCR), the mRNA expression profile is calculated in relative quantity format, followed using an independent sample *t* test (SPSS software) to analyze the statistical difference. Differences were considered significant at *P* < 0.05.

## Supplementary Information

Below is the link to the electronic supplementary material.

Supplementary file1 (DOCX 442 KB)

## Data Availability

The datasets generated during and/or analyzed during the current study are available from the corresponding author on reasonable request.
