# Supplementary material for: Fine-mapping and association analysis of candidate genes for papilla number in sea cucumber, Apostichopus japonicus
Source: Mar Life Sci Technol. 2022 Aug 23;4(3):343–55. doi: 10.1007/s42995-022-00139-w (PMC10077181; doi:10.1007/s42995-022-00139-w)
Supplement: Supplementary file 1 — Supplementary file1 (DOCX 442 KB) [file 42995_2022_139_MOESM1_ESM.docx]

Supplementary materials

**Fine-mapping and association analysis of candidate genes for papilla number in sea cucumber, *Apostichopus japonicus***

**Xinghai Zhu1 · Ping Ni1 · Marc Sturrock2 · Yangfan Wang1 · Jun Ding3 · Yaqing Chang3 · Jingjie Hu4 · Zhenmin Bao1**


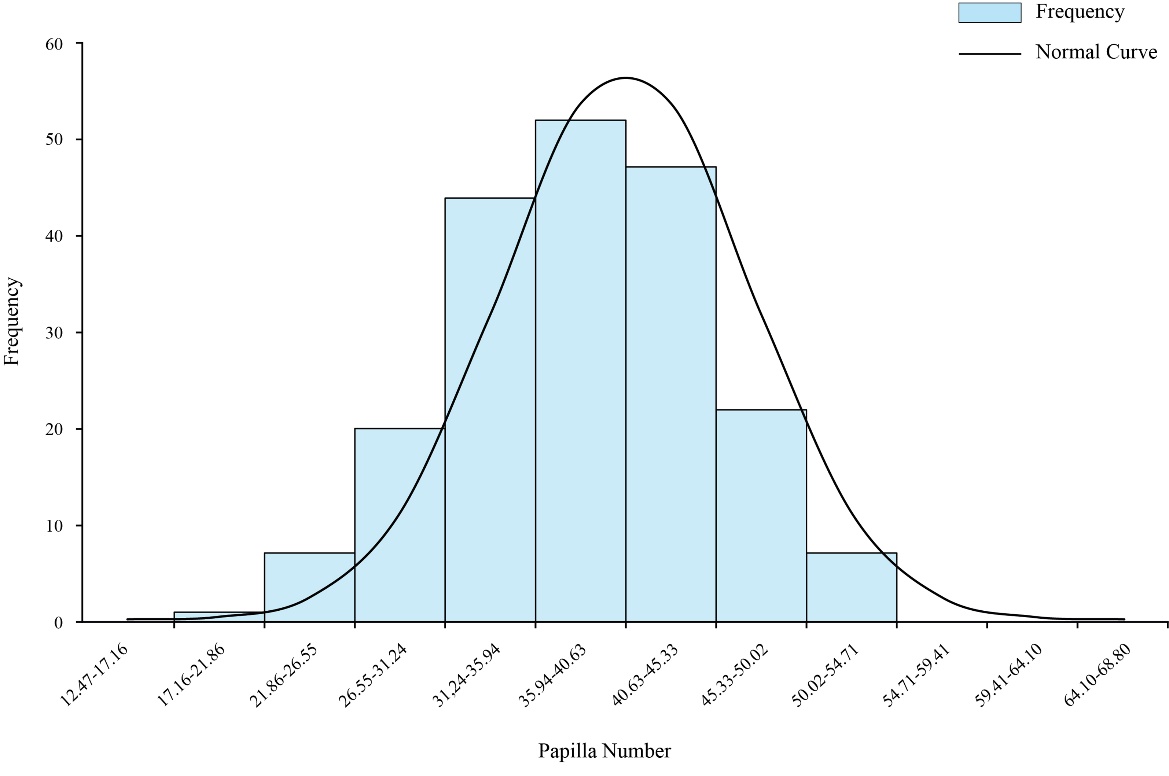


Fig. S1. The distribution of papilla number of 200 sea cucumbers.


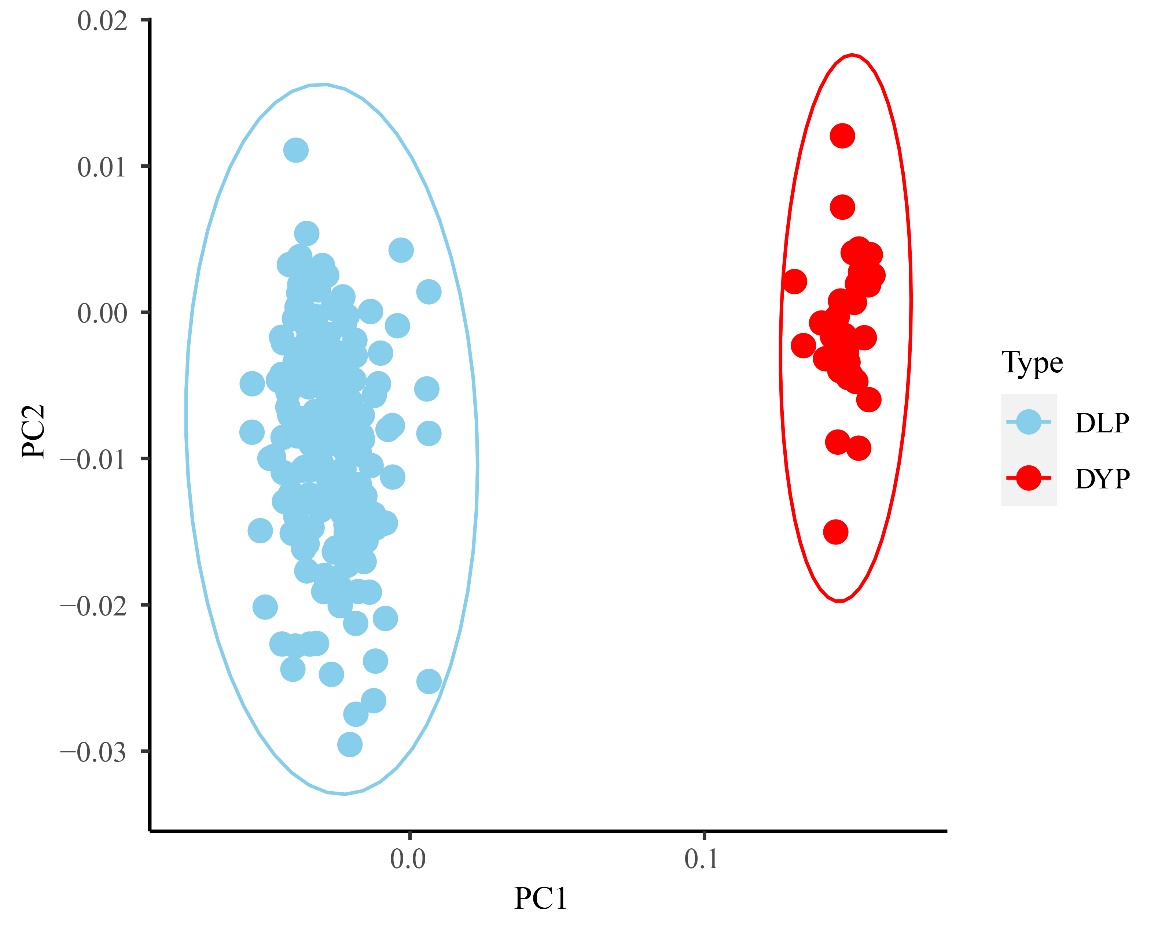


Fig. S2. The principal component analysis of sea cucumbers.


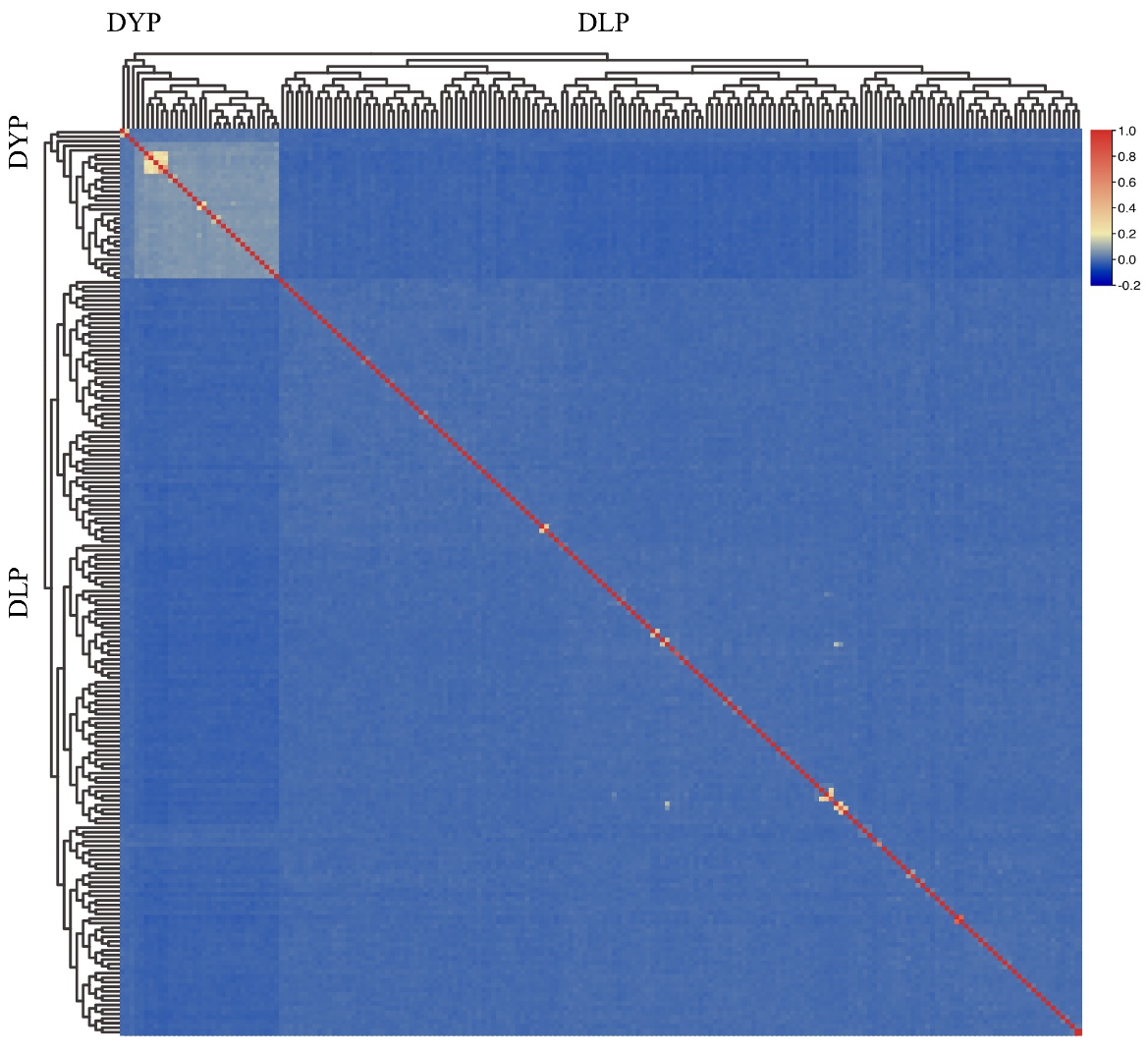


Fig. S3. The kinship structure among the re-sequencing samples.
